# COVID-19 pandemic and health worker stress: The mediating effect of emotional regulation

**DOI:** 10.1371/journal.pone.0259013

**Published:** 2021-11-24

**Authors:** Zoilo Emilio García-Batista, Kiero Guerra-Peña, Vahid Nouri Kandany, María Isabel Marte, Luis Eduardo Garrido, Luisa Marilia Cantisano-Guzmán, Luciana Moretti, Leonardo Adrián Medrano

**Affiliations:** 1 Pontificia Universidad Católica Madre y Maestra (PUCMM), Santiago de los Caballeros, Dominican Republic; 2 Unidad de Investigación de la Escuela de Medicina, Universidad Autónoma de Santo Domingo (UASD), Santiago de los Caballeros, Dominican Republic; 3 Escuela de Medicina, Universidad Tecnológica de Santiago (UTESA), Santiago de los Caballeros, Dominican Republic; 4 Universidad Siglo 21, Córdoba, Argentina; Universidad Autonoma de Madrid, SPAIN

## Abstract

**Background/Introduction:**

Psychological and physical well-being of health personnel has been significantly affected by COVID-19. Work overload and continuous exposure to positive COVID-19 cases have caused them fatigue, stress, anxiety, insomnia and other detriments. This research aims: 1) to analyze whether the use of cognitive reevaluation and emotional suppression strategies decreases and increases, respectively, stress levels of health personnel; 2) to quantify the impact of contact with patients with COVID-19 on stress levels of medical staff.

**Method:**

Emotion regulation strategies (cognitive reevaluation and emotional expression) and stress levels were evaluated in 155 Dominican physicians who were treating people infected with COVID-19 at the moment of the study (67.9% women and 32.1% men; mean age = 34.89; SD = 9.26). In addition, a questionnaire created by the researchers quantified the impact that contact with those infected had on their stress levels.

**Results:**

Contact with patients with COVID-19 predicts increased use of emotion suppression strategies, although is not associated with the use of cognitive reevaluation. These findings lead to an even greater increase in stress on health care providers.

**Conclusions:**

Contextual contingencies demand immediate responses and may not allow health personnel to use cognitive re-evaluation strategies, leaning more towards emotion suppression. However, findings regarding high levels of stress require the implementation of intervention programs focused on the promotion of more functional emotion regulation strategies. Such programs may reduce current stress and prevent post-traumatic symptoms.

## Introduction

Coronavirus Disease 2019 (COVID-19) pandemic continues to spread internationally, putting increased pressure on health care workers due to the risk of exposure to a highly contagious disease, which can be very severe and even lethal for a percentage of the sick. The need to provide immediate responses and the volume of people infected generate an overload of work that increases levels of fatigue and stress [1]. In addition, the risks of exposure, concern about infecting loved ones, self-isolation measures and family-work conflict are factors that all together increase the likelihood of emotional disorders (e.g. general anxiety disorder, major depression disorder, panic disorder) and problems associated with chronic stress [2, 3].

Several studies indicate an increase in the prevalence of mental health symptoms among health workers who treat patients with COVID-19. A study developed in March 2019 with 1257 physicians and nurses indicated that 50.4%, 44.6%, 34.0%, and 71.5% had symptoms of depression, anxiety, insomnia, and distress, respectively [4]. In a previous study during the acute SARS-Cov2 outbreak [2], 89% of health workers reported psychological symptoms and disorders (e.g. including persistent depression, anxiety, panic attacks, psychomotor excitement, psychotic symptoms, delirium, and even suicidality). Sources of distress may include feelings of vulnerability or loss of control and concerns about one’s health, the spread of the virus, the health of family and others, changes in work, and isolation [4].

This situation is even more complex for health professionals in developing countries [5], as is the case in the Dominican Republic. The lack of sufficient resources for patients’ treatment and health worker protection [6] increases the overload of health workers and the risk of experiencing stress-related problems.

Faced with this scenario, various agencies have highlighted the need to address the psychological safety of health workers [7]. As the Pan American Health Organization [8] points out, attending to the mental health and psychosocial well-being of health workers is as important as taking care of their physical health. However, psychological factors that could mediate the levels of psychological stress during the course of the pandemic have not yet been studied empirically.

Within this framework, emotion regulation (ER) strategies may play a significant role. In the last decade, there has been increasing interest in exploring how people manage or regulate their emotions through specific strategies [9]. The model of emotion regulation process is one of the most influential theoretical proposals to outline the mechanisms by which people modulate their emotions. Within this model, two well-defined ER strategies have been empirically explored: Cognitive reevaluation (CR), a cognitive strategy that involves redefining a potentially emotive situation in such a way as to change its emotional impact; and emotion suppression (ES), a form of response modulation that involves inhibiting the expressive behavior of the emotion in progress [10].

In this same line, CR (assigning a "non-emotional" meaning to an event) and ES (controlling the somatic response to an emotion) have been differentially associated with psychological adjustment and health variables, with the negative effects of suppression overlapping with the positive effects of reappraisal [9, 10]. Thus, CR has been positively correlated with self-esteem, optimism, personal growth and purpose in life, while inverse correlations have been reported with the negative effects, stress and depression [9, 11]. On the other hand, ES increases physiological activity and has negative effects on memory, and has been positively associated with negative affect, anxiety and depression [12, 13].

Overall, previous findings lead to the assumption that health personnel who make adequate use of ER strategies will have lower levels of perceived stress. Conversely, those professionals with greater difficulties in regulating their emotions will present greater symptoms associated with stress [14–16].

It is important to analyze the factors involved in the appropriate stress regulation not only because they are relevant for the psychological well-being of health-care workers, but also for the patients themselves. Inadequate stress regulation can diminish the empathy that health personnel may have towards patients, reduce impulse control, increase aggression and, in general, affect the quality of their services. In addition, high levels of stress can lead to health personnel making attentional mistakes, such as medication failure or mistakes in the implementation of patient care techniques [16–18].

Depending on the importance of identifying protective factors of stress in health-care workers, the present study aims to analyze whether ER strategies have a mediating role on perceived stress of health-care workers. More specifically, it is hypothesized that the use of cognitive reevaluation strategies decreases stress levels of health-care workers and that emotion suppression strategies increase stress levels. In addition, this study will quantify the impact of contact with patients with COVID-19 (number of patients and hours spent) and of the team´s perceived safety on medical staff´s stress.

## Methods

This research was reviewed and approved by National Council of Bioethics in Health/ Consejo Nacional de Bioética en Salud (CONABIOS) of the Dominican Republic. The protocol registration number in CONABIOS was -005-2019.

### Participants

The sample was composed of 155 physicians (67.9% women and 32.1% men) from the Dominican Republic, ranging in age from 23 to 66 (Mage = 34.89; *SD* = 9.26). Regarding their civil status, 38.7% reported being single, 42.0% married, 1.9% divorced, and 17.4% cohabiting. Additionally, 38.1% worked in public centers, 40.0% in private centers, 21.3% in both public and private centers, and 0.6% self-managed. In terms of their rank within the health centers, 26.5% were general doctors, 27.7% were specialists, 14.2% were subspecialists, 10.4% were medical residents, 1.3% were medical residency coordinators, 5.8% were department directors, 0.6% were hospital directors, 0.6% were nurses, 12.3% were medical interns, and 0.6% did not specify their ranks. all study participants completed a written informed consent.

### Instruments

#### Perceived Stress Scale (PSS-14)

This instrument was designed by Cohen et al. [19] to measure the degree to which life situations are perceived as stressful during the last month (e.g., In the last month, how often have you been upset because of something that happened unexpectedly?). Its approximate application time is 8–10 min, and it is made up of 14 direct and indirect items (reverse item score). It uses a Likert-type response format of 5 alternatives, with a range from 0 (never) to 4 (very often), inverting the score on items 4, 5, 6, 7, 9, 10 and 13. The scale scores from 0 to 56; higher scores indicate greater perceived stress. This scale has demonstrated in several populations to have consistent psychometric properties for the measurement of stress [20].

#### Emotion Regulation Questionnaire (ERQ) [10]

This instrument is designed to evaluate general emotion regulation strategies by means of 10 items, in detail, 6 items that assess cognitive reevaluation (e.g. When I want to feel less negative emotion (such as sadness or anger), I change what I’m thinking about) and 4 items that represent the suppression of emotional expression (e.g. I control my emotions by not expressing them). These are evaluated by a Likert-type scale with 7 response options ranging from 1 (totally disagree), 2 (disagree), 3 (slightly disagree), 4 (neither agree nor disagree), 5 (slightly agree), 6 (agree), to 7 (totally agree) [21–23].

#### COVID-19 patient contact

A questionnaire was designed for this study to measure the amount of COVID-19 patient contact. Two questions were included to measure the amount of COVID-19 patient contact: “What is the number of hours per day that you work in contact with people with Covid-19?” and “How many people with Covid-19 do you see on average per day?”.

#### COVID-19 perceived equipment safety

A question was designed for this study to measure the degree of perceived safety regarding the equipment used: “In case it applies to your case, please rate from 1 (not at all safe) to 10 (completely safe) how safe you feel with the protective equipment against the New Coronavirus (Covid-19) that you have at your disposal in the health center where you work”.

### Statistical analyses

#### Modelling specifications

Structural equation modeling (SEM) was used to assess the mediating effects of emotion regulation on the effects of COVID-19 patient contact and the perception of equipment safety on the perceived stress of the healthcare workers. The latent variable corresponding to COVID-19 patient contact had two observed indicators: “What is the number of hours per day that you work in contact with people with Covid-19?” and “How many people with Covid-19 do you see on average per day?”. Because the model contained a mixture of continuous and categorical variables, the weighted minimum squares with mean- and variance-adjusted standard errors (WLSMV) estimator was employed, which is widely recommended for models that include ordinal-categorical variables [24]. As the underlying structure of the scores from the Emotion Regulation Questionnaire items is composed of two factors (cognitive reevaluation and emotion suppression), they were estimated using an exploratory structural equations model (ESEM) [25], with factors rotated using the Geomin algorithm [26]. In general, psychological measures are fallible or impure indicators of their underlying trait, and as such, the factor structures containing them are more accurately estimated with unrestricted models that allow the items to load freely on different factors [27, 28]. Nevertheless, if the factorial structure underlying the emotion regulation items truly conformed to an independent clusters model, this could be detected in the ESEM model as all the cross-loadings would be non-significant or of trivial magnitude. If that were the case, the ESEM model could then be replaced by a confirmatory factor analysis (CFA) model.

The significance and confidence intervals of the indirect effects was evaluated using bootstrapping, which has demonstrated optimal functioning [29]. A total of 50,000 random samples with replacement were generated from the empirical data, and the 95% confidence intervals were constructed by taking the values corresponding to 2.5 and 97.5 percentiles of the parameter estimate distribution. In order to combine bootstrapping with ESEM factors, the ESEM within CFA method was employed [28]. Also, to evaluate the size of the mediation effects, Cohen’s [30] benchmarks of .01 for small, .09 for medium, and .25 for large effects were used for the completely standardized indirect effects (*ab*_*cs*_) [31].

Wording effects resulting from the Perceived Stress Scale have been balanced, with half the items reversed coded, were modeled using random intercept item factor analysis (RIIFA) [32]. The RIIFA model adds a wording method factor where the pro-trait items have loadings of +1 and the *recoded* reversed items have loadings of -1. Thus, it posits an artifactual relationship between the groups of items that contrasts with the substantive factor, where all the items are expected to have loadings of the same sign. Additionally, the wording factor was specified to be uncorrelated with the substantive factor in order to ensure identification. The RIIFA model has performed well in accounting for wording variance arising from the responses to scales that combine items of opposite polarity [33, 34]. It should be noted that if there is no wording variance in the data this would be reflected in the loadings on the RIIFA factor, which would be non-significant or of trivial magnitude. If that were the case, the RIIFA factor could be eliminated from the model.

#### Fit criteria

The fit of the SEM model was assessed with four complimentary indices: the comparative fit index (CFI), the Tucker-Lewis index (TLI), the root mean square error of approximation (RMSEA), and the standardized root mean square residual (SRMR). Values of CFI/TLI greater than or equal to .90 and .95 have been suggested that reflect acceptable and excellent fits to the data, while values of RMSEA less than .08 and .05 may indicate reasonable and close fits to the data, respectively [35–37]. In the case of SRMR, a value less or equal to .08 has been found to indicate a good fit to the data [35, 38]. It should be noted that because the values of these fit indices are also affected by incidental parameters not related to the size of the misfit [39–41], they should not be considered golden rules, and must be interpreted with caution [36, 42].

#### Reliability analyses

The internal consistency reliability of the psychological scale scores was evaluated with Green and Yang’s [43] categorical omega coefficient. Categorical omega takes into account the ordinal nature of the data to estimate the reliability of the observed scores, and as such, it is recommended for Likert-type item scores [44, 45]. In order to provide common reference points with the previous literature, Cronbach’s [46] alpha with the items treated as continuous was also computed and reported. Additionally, the reliability of the scores for COVID-19 patient contact, which were derived from two continuous measures, was estimated using alpha based on the standardized scores [47]. For all coefficients 95% confidence intervals were computed across 1,000 bootstrap samples using the bias-corrected and accelerated approach [48].

#### Missing data handling

Missing data for the variables included in the SEM model was very small, with only a 0.3% total missing value rate. None of the items from the Perceived Stress Scale had missing values, while two items from the Emotion Regulation Questionnaire had one missing value each (0.6% rate). Neither age, sex, or the two COVID-19 patient contact items had missing values. Finally, the variable measuring the perceived safety provided by the protective equipment had 7.7% cells with missing values. According to Little’s [49] MCAR test the data were missing completely at random (χ^2^ = 105.11, *df* = 84, *p* = .059). Due to the very small amount of missingness and the MCAR mechanism, the missing data was handled using pairwise deletion [50].

#### Analysis software

Data handling, descriptive statistics, and Little’s MCAR test were computed using the IBM SPSS software version 25. Sample correlations and the SEM model were estimated with the *Mplus* program version 8.3. Internal consistency reliability with the categorical omega and alpha coefficients was estimated with the *ci*.*reliability* function contained in the *MBESS* package (version 4.6.0) [51].

## Results

Health-care professionals that participated in this study worked an average of 4.49 hours daily (SD = 4.17) with COVID-19 patients and had daily contact with an average of 2.46 people (SD = 3.81) infected with the virus. Regarding the perceived safety provided by their protective equipment, the mean scores were 3.35 (SD = 2.66) on the 1–10 response scale. Additionally, the mean scores were 1.78 (SD = 0.64) across the perceived stress items (0–4 scale), 3.45 (SD = 0.79) across the cognitive reevaluation items (1–5 scale), and 2.93 (SD = 1.05) across the emotion suppression items (1–5 scale). The sample correlations between the observed variables included in the SEM model are presented in S1 Table.

According to the categorical omega reliability coefficient, all the scales had adequate internal consistency reliability. In the case of the perceived stress scores, the reliability estimate was .928 (95% CI = .894, .943). For the cognitive reevaluation scale, the reliability estimate was .723 (95% CI = .595, .794), while for emotional suppression it was .762 (95% CI = .663, .822). In order to provide a common reference with previous studies using these measures, the (suboptimal) alpha estimates for these scales’ scores were: .898 (95% CI = .873, .919) for perceived stress, .682 (95% CI = .582, .760) for cognitive reevaluation and .749 (95% CI = .665, .815) for emotion suppression. Finally, the COVID-19 patient contact composite score (daily number of hours treating COVID-19 patients *and* daily number of COVID-19 patients treated) had a reliability of .639 (95% CI = .399, .743) according to the alpha coefficient.

**Fig 1:**
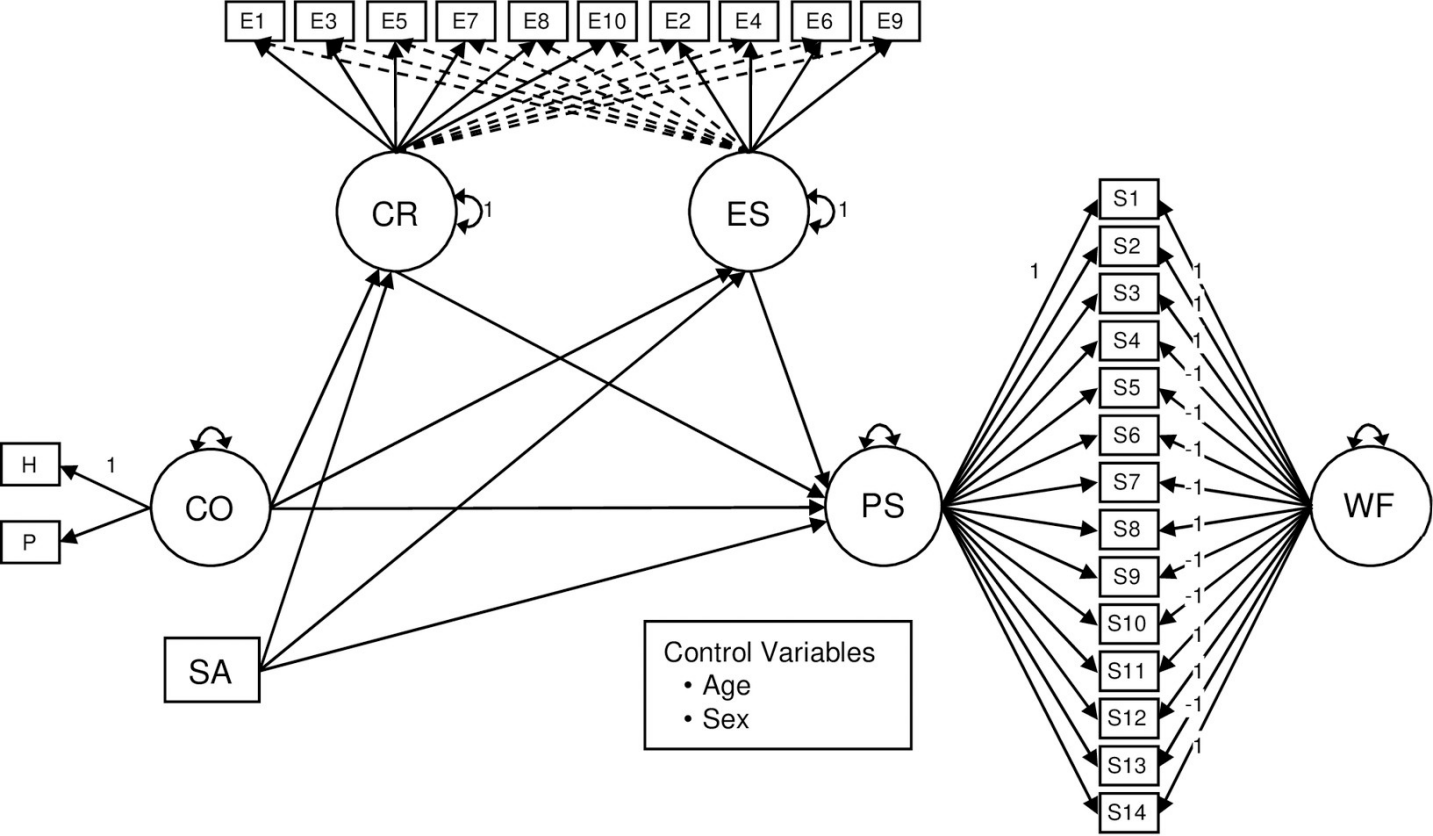
SEM model evaluating the mediating effects of emotional regulation on the impact of COVID-19 contact and equipment safety on the perceived stress of medical personnel. *Note*. CO = contact with COVID-19 patients; SA = perceived safety provided by the protective equipment; PS = perceived stress; CR = cognitive regulation; ES = emotional suppression; WF = wording factor; H = daily number of hours treating COVID-19 patients; P = daily number of COVID-19 patients treated; S1-S14 = Perceived Stress Scale items; E1-E10 = Emotional Regulation Questionnaire items. Squares represent observed variables. Circles represent latent variables. Full unidirectional arrows linking circles and rectangles represent the target factor loadings. Dotted unidirectional arrows linking circles and rectangles represent the cross-loadings. Bidirectional arrows connecting a single circle represent the factor variances. For simplicity, the control variables do not appear represented in the model, as well as the item uniquenesses, the factor uniquenesses, and the residual correlations between variables that share the same predictors.

Fig 1 depicts a simplified version of the estimated SEM model that assessed the mediating effects on the relationship of COVID-19 patient contact and equipment safety to perceived stress of medical personnel. In order to statistically control for age and sex in the SEM model (Fig 1), all the variables, except the RIIFA wording factor for perceived stress (where the pro-trait and recoded reversed items have loadings of 1 and -1, respectively), were regressed on them. Also, because age and sex were exogenous variables, they were allowed to correlate. As typical, the residuals from endogenous variables that shared the same predictors were allowed to correlate.

According to the different indices evaluated, the fit of the estimated SEM model was good: *χ*^2^_350_ = 515.43 (*p* < .001), CFI = .944, TLI = .935, RMSEA = .055 (90% CI = .045, .065), and SRMR = .063. The item factor loadings derived from the SEM model are presented in Table 1. In general, the factors had adequately sized factor loadings. The mean factor loadings were .68 for the perceived stress factor, .58 for cognitive reevaluation, .63 for emotion suppression, and .69 for COVID-19 patient contact. As expected, the items from the Emotion Regulation Questionnaire produced several significant cross-loadings of considerable magnitude, supporting the use of ESEM modeling for the two factors derived from this instrument. On the other hand, the loadings on the wording method factor were significant and of moderate magnitude (.233), revealing the presence of wording variance in the perceived stress item scores.

**Table 1:** Item factor loadings for the estimated SEM model.

	Factors				
Item	CO	PS	WF	CR	ES
H	.594**				
P	.791**				
S1		.715**	.233**		
S2		.792**	.233**		
S3		.816**	.233**		
S4		.718**	-.233**		
S5		.752**	-.233**		
S6		.698**	-.233**		
S7		.673**	-.233**		
S8		.567**	-.233**		
S9		.802**	-.233**		
S10		.761**	-.233**		
S11		.734**	-.233**		
S12		.255**	-.233**		
S13		.429**	-.233**		
S14		.754**	-.233**		
E1				.577**	-.007
E3				.416**	.088
E5				.535**	-,499**
E7				.616**	.255*
E8				.646**	.271*
E10				.685**	-.079
E2				-.062	.792**
E4				.008	.595**
E6				.168	.692**
E9				.290**	.449**

Note. Co = contact with COVID-19 patients; PS = perceived stress; WF = Wording factor; Cr = Cognitive regulation; ES = Emotional suppression; H = Daily number of hours treating COVID-19 patients; P = daily number of COVID-19 patients treated; S1-S14 = Perceived Stress Scale Items; E1-E10 = Emotion Regulation Questionnaire Items.

*p < .05

**p < .01.

The standardized direct and indirect regression weights (β), residual correlations, and correlations from the estimated SEM model are shown in Table 2. The main findings from the results included in the table are: *first*, COVID-19 patient contact increased emotion suppression (β = .363, *p* = .002) of the medical personnel, but not their cognitive reevaluation (β = .124, *p* = .384). *Second*, cognitive reevaluation was associated with perceived stress (β = -.425, *p* < .001), whereas emotion suppression increased it (β = .645, *p* < .001). *Third*, emotion suppression mediated the effects of COVID-19 patient contact with a near large effect size (β = .234, *p* < .01), but cognitive reevaluation was not a significant mediator (β = -.053, *p* > .05). *Fourth*, COVID-19 patient contact did not have a direct effect on perceived stress (β = .122, *p* = .183). *Fourth*, as the perceived safety provided by the protective equipment did not affect emotion suppression (β = -.133, *p* = .209), cognitive reevaluation (β = .041, *p* = .653), or perceived stress (β = .083, *p* = .302) of the personnel, neither cognitive reevaluation (β =.-.018, *p* > .05) nor emotion suppression (β =.-.086, *p* > .05) were significant mediators in relation to this variable. *Sixth*, older workers had less COVID-19 patient contact (β = -.238, *p* = .025), and reported more cognitive reevaluation (β = .269, *p* = .003). *Seventh*, males reported less cognitive reevaluation (β = -.510, *p* < .001) and less emotion suppression (β = -.235, *p* = .023) than females. *Finally*, the proportion of variance explained by the predictors of the mediators and dependent latent variables were: .244 (*p* < .001) for cognitive reevaluation, .243 (*p* = .002) for emotion suppression and .578 (*p* < .001) for perceived stress.

**Table 2:** Regressions weights and correlations from the estimated SEM model.

*Effect*	Standardized solution			
Variables	Estimate	SE	*p*	95% CI
*Effects from CO to PS*				
Total	.304**	-	< .01	.094, .548
Total indirect	.182	-	> .05	-.037, .449
Specific indirect				
CO → CR → PS	-.053	-	> .05	-.283, .062
CO → ES → PS	.234**	-	< .01	.083, .503
Direct				
CO → PS	.122	.092	.183	-.058, .302
*Effects from SA to PS*				
Total	-.020	-	> .05	-.265, .191
Total indirect	-.104	-	> .05	-.342, .057
Specific indirect				
SA → CR → PS	-.018	-	> .05	-.129, .110
SA → ES → PS	-.086	-	> .05	-.322, .059
Direct				
SA → PS	.083	.081	.302	-.076, .242
*Remaining direct effects*				
CR → PS	-.425	.105	.000	-.631, -.219
ES → PS	.645	.086	.000	.476, .814
CO → CR	.124	.142	.384	-.154, .402
CO → ES	.363**	.115	.002	.138, .588
SA → CR	.041	.092	.653	-.139, .221
SA → ES	-.133	.106	.209	-.341, .075
Age → CO	-.238*	.107	.025	-.448, -.028
Age → SA	.078	.117	.508	-.151, .307
Age → CR	.269**	.090	.003	.093, .445
Age → ES	-.096	.095	.313	-.282, .090
Age → PS	-.099	.080	.214	-.256, .058
Sex → CO	-.002	.097	.982	-.192, .188
Sex → SA	.076	.098	.438	-.116, .268
Sex → CR	-.510**	.067	.000	-.641, -.379
Sex → ES	-.235*	.103	.023	-.437, -.033
Sex → PS	.041	.070	.558	-.096, .178
*Residual correlations*				
CO ⟷ SA	.349**	.089	.000	.175, .523
CR ⟷ ES	.154	.139	.269	-.118, .426
*Control correlations*				
Age ⟷ Sex	.375**	.068	.000	.242, .508

*Note*. CO = contact with COVID-19 patients; PS = perceived stress; CR = cognitive regulation; ES = emotional suppression; SA = perceived safety provided by the protective equipment; SE = standard error; CI = confidence interval. The coding for the variable sex was 0 for females and 1 for males. The significance of the indirect effects was evaluated with bootstrapping.

*p < .05

** *p* < .01.

## Discussion

COVID-19 has a great impact on the physical and mental health of millions of people [4]. Concern about infection or transmission to a family member, social isolation, and economic impact have led to an increase in the prevalence of stress-related problems in the general population [52]. However, the impact of this pandemic on stress is especially critical for health-care workers. The consequences of high and chronic stress are multiple among workers, even more on healthcare professionals that are exposed in daily basis to highly contagious and lethal virus. First, it affects the mental health of workers, as suffering from occupational stress doubles the probability of developing a mental disorder [53, 54], and predicts the development of anxious and depressive clinical symptoms [55]. Secondly, it is associated with the development of physical diseases, such as cardiovascular problems [14–16, 56]. Finally, stress can diminish the empathy of health-care professionals towards patients, reduce their impulse control and affect the quality of their services [16–18].

This situation is even more critical in countries with fewer health resources [5], such as the Dominican Republic. The lack of sufficient resources for the treatment of patients and for the protection of health-care workers [6] increases the overload of them and the risk of experiencing problems associated with stress. On the other hand, it leads to the need for health-care professionals to make ethically and morally difficult decisions about those who receive these scarce resources. Their decisions can mean life or death for many. This can cause chronic stress, moral damage, and feelings of guilt [57].

Within this context, the identification of protective psychological factors that allow health-care professionals to reduce their stress levels and protect their mental health is critical. The results obtained in this study support the adjustment of a mediational model, where emotion regulation (ER) strategies play an important role on perceived stress levels.

ER refers to a set of processes aimed to modulate the emotional state in order to respond to a series of external demands in an appropriate way [9]. Results indicate that when exposed to contact with patients with COVID-19, health-care workers tend to use predominantly strategies of emotion suppression. In this regard, it should be noted that they probably have no other alternative, since faced with the need to give an immediate response; doctors make a deliberate effort to limit emotion expression behaviors. Unfortunately, as results indicate, using this type of strategy increases stress levels.

In addition, previous research with refugees or people who were exposed to traumatic situations indicates that the use of emotion suppression strategies predicts the development of post-traumatic symptoms and increases the likelihood of developing mental problems in the future [58]. Furthermore, some studies suggest that emotion suppression is an aggravating factor in the effects of traumatic experience [59].

It is likely that contextual contingencies (e.g., urgency and speed of patient care, number of patients to be seen) will not allow health-care professionals to make use of cognitive reevaluation strategies. As results indicate, contact with patients with COVID-19 predicts increased use of emotion suppression strategies, but is not associated with the use of cognitive reevaluation, which is shown to be inversely associated with stress levels.

These findings allow us to affirm that health-care workers are not only exposed to strong stressors, but that these environmental contingencies do not favor the deployment of more functional strategies of emotional regulation either.

For this reason, it is important that health-care workers receive support and containment through intervention programs focused on promoting more functional ER strategies [9, 60]. The aim would not be to avoid the use of emotion suppression, as this is probably the most appropriate strategy for dealing with these situational contingencies. Rather, the goal should be to promote a flexible use of emotion regulation strategies, which decreases stress levels and the likelihood of developing post-traumatic symptoms. One avenue to consider for future research is the potential moderation effect of perceived safety provided by the protective equipment regarding the relationship between COVID-19 patient contact and perceived stress, or in terms of the relationship between COVID-19 patient contact and the emotion regulation strategies.

Based on all the above findings, it is imperative to develop measures and programs aimed at improving the mental health of health-care workers. This should be done as soon as possible, since disfunctional emotion regulation not only puts at risk the psychological well-being of health-care professionals, but also patients’ health. When health-care workers are under great stress, they can make potentially fatal treatment failures [16–18]. The need for an immediate response is even greater when one considers that stress and emotional instability may result in lost workdays that would further limit the human resources that currently assist patients with COVID-19, making them a potential danger if a second wave of infection occurs.

## Supporting information

S1 Table Sample correlations between the variables included in the SEM mediation model. (DOCX)

S1 File Database. (SAV)
