# Supplementary material for: COVID-19 pandemic and health worker stress: The mediating effect of emotional regulation
Source: PLoS One. 2021 Nov 24;16(11):e0259013. doi: 10.1371/journal.pone.0259013 (PMC8612545; doi:10.1371/journal.pone.0259013)
Supplement: S1 Table — (DOCX) [file pone.0259013.s001.docx]

Table A1

*Sample correlations between the variables included in the SEM mediation model*

| Var | Age | Sex | H | P | SA | S1 | S2 | S3 | S4 | S5 | S6 | S7 | S8 | S9 | S10 | S11 | S12 | S13 | S14 | E1 | E2 | E3 | E4 | E5 | E6 | E7 | E8 | E9 | E10 |
| --- | --- | --- | --- | --- | --- | --- | --- | --- | --- | --- | --- | --- | --- | --- | --- | --- | --- | --- | --- | --- | --- | --- | --- | --- | --- | --- | --- | --- | --- |
| Age | 1.0 |  |  |  |  |  |  |  |  |  |  |  |  |  |  |  |  |  |  |  |  |  |  |  |  |  |  |  |  |
| Sex | .38 | 1.0 |  |  |  |  |  |  |  |  |  |  |  |  |  |  |  |  |  |  |  |  |  |  |  |  |  |  |  |
| H | -.23 | -.01 | 1.0 |  |  |  |  |  |  |  |  |  |  |  |  |  |  |  |  |  |  |  |  |  |  |  |  |  |  |
| P | -.14 | -.11 | .47 | 1.0 |  |  |  |  |  |  |  |  |  |  |  |  |  |  |  |  |  |  |  |  |  |  |  |  |  |
| SA | .11 | .11 | .06 | .35 | 1.0 |  |  |  |  |  |  |  |  |  |  |  |  |  |  |  |  |  |  |  |  |  |  |  |  |
| S1 | -.20 | -.06 | .27 | .29 | .04 | 1.0 |  |  |  |  |  |  |  |  |  |  |  |  |  |  |  |  |  |  |  |  |  |  |  |
| S2 | -.22 | .03 | .06 | .19 | .13 | .65 | 1.0 |  |  |  |  |  |  |  |  |  |  |  |  |  |  |  |  |  |  |  |  |  |  |
| S3 | -.19 | .01 | .13 | .15 | .11 | .72 | .71 | 1.0 |  |  |  |  |  |  |  |  |  |  |  |  |  |  |  |  |  |  |  |  |  |
| S4 | -.23 | -.10 | .15 | .13 | .07 | .39 | .56 | .51 | 1.0 |  |  |  |  |  |  |  |  |  |  |  |  |  |  |  |  |  |  |  |  |
| S5 | -.25 | .00 | .23 | .14 | -.05 | .39 | .51 | .50 | .70 | 1.0 |  |  |  |  |  |  |  |  |  |  |  |  |  |  |  |  |  |  |  |
| S6 | -.22 | .02 | .17 | .17 | .08 | .38 | .51 | .56 | .49 | .63 | 1.0 |  |  |  |  |  |  |  |  |  |  |  |  |  |  |  |  |  |  |
| S7 | -.27 | .02 | .14 | .16 | .00 | .42 | .39 | .57 | .50 | .53 | .48 | 1.0 |  |  |  |  |  |  |  |  |  |  |  |  |  |  |  |  |  |
| S8 | -.16 | -.09 | .13 | .18 | -.06 | .40 | .46 | .53 | .37 | .27 | .33 | .33 | 1.0 |  |  |  |  |  |  |  |  |  |  |  |  |  |  |  |  |
| S9 | -.21 | -.02 | .21 | .18 | .06 | .55 | .66 | .54 | .63 | .64 | .59 | .54 | .42 | 1.0 |  |  |  |  |  |  |  |  |  |  |  |  |  |  |  |
| S10 | -.25 | .03 | .10 | .20 | .09 | .40 | .62 | .51 | .55 | .64 | .55 | .62 | .28 | .74 | 1.0 |  |  |  |  |  |  |  |  |  |  |  |  |  |  |
| S11 | -.25 | .01 | .28 | .16 | .01 | .60 | .58 | .65 | .43 | .49 | .58 | .46 | .52 | .51 | .46 | 1.0 |  |  |  |  |  |  |  |  |  |  |  |  |  |
| S12 | -.40 | -.15 | .13 | .03 | -.12 | .24 | .13 | .21 | -.01 | .17 | .12 | .12 | .04 | .07 | .09 | .28 | 1.0 |  |  |  |  |  |  |  |  |  |  |  |  |
| S13 | -.12 | .09 | .05 | .14 | .02 | .31 | .36 | .28 | .24 | .31 | .29 | .31 | .29 | .26 | .46 | .26 | .14 | 1.0 |  |  |  |  |  |  |  |  |  |  |  |
| S14 | -.23 | -.14 | .22 | .27 | .00 | .58 | .59 | .61 | .52 | .53 | .46 | .41 | .56 | .53 | .48 | .60 | .35 | .30 | 1.0 |  |  |  |  |  |  |  |  |  |  |
| E1 | .13 | -.20 | .02 | .06 | .02 | -.02 | -.11 | -.11 | -.10 | -.13 | -.09 | -.26 | -.18 | -.13 | -.24 | -.12 | .01 | -.16 | -.02 | 1.0 |  |  |  |  |  |  |  |  |  |
| E2 | -.23 | -.26 | .18 | .24 | .03 | .36 | .33 | .46 | .34 | .31 | .28 | .30 | .31 | .41 | .34 | .33 | .21 | .24 | .47 | .16 | 1.0 |  |  |  |  |  |  |  |  |
| E3 | .07 | -.11 | -.07 | .06 | .10 | -.03 | -.05 | .06 | -.06 | -.03 | .02 | -.12 | -.01 | -.10 | -.09 | .07 | .15 | -.28 | .08 | .33 | .17 | 1.0 |  |  |  |  |  |  |  |
| E4 | -.13 | -.18 | .14 | .15 | -.12 | .23 | .28 | .24 | .20 | .31 | .16 | .06 | .32 | .22 | .15 | .20 | .14 | .24 | .36 | .09 | .53 | -.07 | 1.0 |  |  |  |  |  |  |
| E5 | .11 | -.03 | .05 | .01 | .02 | -.28 | -.32 | -.45 | -.29 | -.36 | -.38 | -.50 | -.24 | -.26 | -.30 | -.39 | -.08 | -.22 | -.21 | .24 | -.26 | .05 | -.16 | 1.0 |  |  |  |  |  |
| E6 | -.19 | -.22 | .21 | .21 | -.08 | .29 | .25 | .26 | .37 | .37 | .32 | .18 | .21 | .27 | .24 | .18 | .19 | .05 | .36 | .17 | .51 | .25 | .50 | -.08 | 1.0 |  |  |  |  |
| E7 | -.08 | -.50 | .06 | .06 | .05 | .04 | .03 | .09 | .06 | .00 | .08 | -.11 | -.02 | -.15 | -.11 | .04 | .13 | -.11 | .04 | .49 | .25 | .31 | .24 | .11 | .28 | 1.0 |  |  |  |
| E8 | -.07 | -.31 | .09 | .13 | .00 | .10 | .00 | .09 | .15 | .08 | .03 | -.10 | -.11 | -.07 | -.01 | .12 | .07 | -.18 | .04 | .35 | .20 | .35 | .10 | .14 | .41 | .60 | 1.0 |  |  |
| E9 | -.18 | -.19 | .19 | .28 | -.01 | .14 | .13 | .20 | .24 | .09 | .01 | .03 | .14 | .04 | .06 | .03 | .12 | -.02 | .12 | .22 | .44 | .18 | .44 | -.02 | .52 | .24 | .32 | 1.0 |  |
| E10 | .06 | -.24 | -.03 | .09 | .04 | -.12 | -.16 | -.09 | -.09 | -.23 | -.10 | -.34 | -.19 | -.23 | -.20 | -.17 | -.21 | -.19 | -.19 | .22 | -.01 | .34 | .08 | .30 | .21 | .32 | .54 | .26 | 1.0 |
| *Note*. H = daily number of hours treating COVID-19 patients; P = daily number of COVID-19 patients treated; SA = perceived safety provided by the protective equipment; S1-S14 = Perceived Stress Scale items; E1-E10 = Emotional Regulation Questionnaire items. The coding for the variable sex was 0 for females and 1 for males. Items S1-S14 and E1-E10 are treated as ordinal-categorical and the correlations between them are polychoric correlations. The remaining correlations including any of the rest of the variables are Pearson correlations. Non-significant correlations (*p* ≥ .05) appear underlined. | | | | | | | | | | | | | | | | | | | | | | | | | | | | | |
